# Uptake and Transport of Monotropein and Monotropein Esters in a Caco‐2/HT29‐MTX‐E12 Co‐Culture Model

**DOI:** 10.1002/mnfr.70571

**Published:** 2026-07-30

**Authors:** Christian Zielinski, Victor Schmalle, Luise A. Lauer, Tim Hammerschick, Felix Rüttler, Walter Vetter, Jan Frank, Felipe Jiménez‐Aspee

**Affiliations:** ^1^ Department of Food Biofunctionality (140b) Institute of Nutritional Sciences University of Hohenheim Stuttgart Germany; ^2^ Department of Food Chemistry (170b), Institute of Food Chemistry University of Hohenheim Stuttgart Germany

**Keywords:** Caco‐2, HT29‐MTX‐E12, intestinal absorption, iridoid, monotropein, permeability

## Abstract

Iridoids are dietary phytochemicals with diverse bioactivities, but their intestinal absorption remains poorly understood. We investigated the uptake, transepithelial transport, and early metabolism of monotropein and two esters (monotropein‐10‐coumarate and monotropein‐10‐cinnamate) using a validated Caco‐2:HT29‐MTX‐E12 co‐culture model. In silico analysis showed high polarity (>186 Å^2^) and predominant ionization at intestinal pH (p*K*_a_ ∼ 4.10–4.14), consistent with low passive permeability. Cellular uptake was rapid and largely time‐independent. Monotropein uptake increased at 4°C versus 37°C, suggesting reduced efflux or metabolism rather than active transport; esters were unaffected. Transepithelial transport was low but increased at apical pH 6.0 compared to pH 7.4. Parallel artificial membrane assays (PAMPA) confirmed low passive permeability. Transporter inhibition indicated partial involvement of sodium–glucose cotransporter 1 (SGLT1) and organic anion transporting polypeptides (OATP) in monotropein uptake. Untargeted HPLC‐MS detected no metabolites, and esters were not hydrolyzed. Overall, permeability was limited and not improved by esterification, suggesting low systemic exposure and a potential role for colonic and hepatic metabolism in vivo.

## Graphical abstract

Monotropein and its naturally occurring ester derivatives (monotropein‐10‐coumarate and monotropein‐10‐cinnamate) from *Gaultheria* spp. berries exhibit limited intestinal permeability in a validated Caco‐2:HT29‐MTX‐E12 co‐culture model. Absorption occurs predominantly via passive diffusion, with partial SGLT1‐ and OATP‐mediated uptake contribution for monotropein only. Esterification progressively increased lipophilicity but did not enhance epithelial permeability, likely due to a molecular size penalty offsetting any lipophilicity gain. No efflux pump involvement (P‐gp, BCRP, MRP), phase I/II epithelial metabolism, or ester hydrolysis was detected. The pH‐dependent permeability of monotropein is consistent with its p*K*_a_ (∼4.14) and the predicted 25‐fold increase in neutral fraction at apical pH 6.0 versus 7.4. Overall, systemic exposure following dietary intake is likely modest, with colonic microbial metabolism and hepatic biotransformation expected to play important roles in vivo.

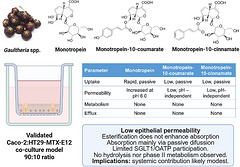

**Abbreviations**
BCA: Bicinchoninic acid
BCRP: Breast cancer resistance protein
BSA: Bovine serum albumin
CCC: Counter‐current chromatography
CYP450: Cytochrome P450
DMSO: Dimethyl sulfoxide
EtOAc: Ethyl acetate
GI: Gastrointestinal
GLUT2: Glucose transporter protein 2
HBSS: Hanks' balanced salt solution
HPLC: High‐performance liquid chromatography
iLOGP: Iterative logarithm of the partition coefficient
Log *P_o_*_/_ *_w_*
: Octanol–water partition coefficient
Log S: Logarithm of aqueous solubility
MCT: Monocarboxylic acid transporter
MeOH: Methanol
MES: 2‐(*N*‐morpholino)ethanesulfonic acid, sodium salt
MRP: Multidrug resistance‐associated protein
MS: Mass spectrometry
MTT: 3‐(4,5‐Dimethylthiazol‐2‐yl)‐2,5‐diphenyltetrazolium bromide
MW: Molecular weight
OATP: Organic anion transporting polypeptide
PAMPA: Parallel artificial membrane permeability assay
Papp: Apparent permeability coefficient
P‐gp: P‐glycoprotein
SGLT1: Sodium‐glucose transport protein 1
TEER: Transepithelial electrical resistance
TPSA: Topological polar surface area
UWL: Unstirred water layer

## 1 Introduction

Iridoids are monoterpenes widely distributed across plant families, including Scrophulariaceae, Rubiaceae and Ericaceae, where they are often found in seeds, roots, stems, leaves, and fruits [1]. Structurally, they are characterized by a bicyclic, H‐5/H‐9β, β‐cis cyclopentane‐pyran system [2]. Berries from *Vaccinium* and *Gaultheria* (Ericaceae), such as blueberries and teaberries, represent important dietary sources. While iridoids have protective functions in plants, they have been associated with diverse bioactivities in humans, including anti‐inflammatory, antioxidant, and cytoprotective effects [1, 3]. Because such activities depend strongly on the intestinal uptake and systemic distribution, understanding their absorption is critical to bridge in vitro bioactivity with potential in vivo efficacy.

**FIGURE 1:**
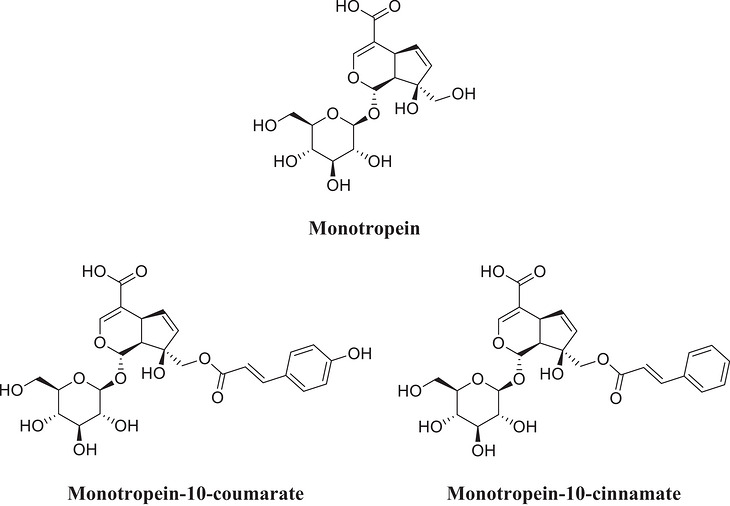
Chemical structures of monotropein, monotropein‐10‐*trans*‐coumarate, and monotropein‐10‐*trans*‐cinnamate.

Among iridoids, monotropein is distinguished by a carboxylic acid moiety at C4 and hydroxyl/hydroxymethyl groups at C7 (Figure 1). In vitro and in vivo studies have demonstrated its anti‐inflammatory and cytoprotective activities [4]. In *Gaultheria phillyreifolia* and *Gaultheria poeppigii* berries, ester derivatives, namely monotropein‐10‐coumarate and monotropein‐10‐cinnamate, have been identified (Figure 1) [5, 6]. Esterification is a common strategy to increase lipophilicity and membrane permeability [7], although the relationship between lipophilicity and permeability is not always linear for large polar glycosides. Phenolic acid conjugates sometimes enhance the anti‐inflammatory and cytoprotective effects in intestinal cells [8, 9]. Our previous work has shown that monotropein and its esters remain partially stable during in vitro gastrointestinal digestion and are efficiently released from the food matrix, suggesting potential for intestinal absorption [6].

The intestinal epithelium is the critical interface governing the absorption and bioavailability of dietary xenobiotics, acting both as a barrier and as a site of biotransformation [10]. Enzymatic phase I and II reactions, transporter proteins and microbial metabolism further influence their fate [11]. Previous studies on other iridoids show variable permeability and transporter interactions, as recently reviewed [1]. Loganin showed high permeability in Caco‐2 cells, involving both passive diffusion and active efflux [12]. Morroniside displayed pH‐dependent transport [13], and geniposide was transported mainly by passive diffusion ex vivo in rat duodenum and jejunum, with evidence of P‐glycoprotein‐mediated efflux [14]. The oral bioavailability of monotropein in rats is low (2%–4%), with tissue distribution to the gut and central nervous system [15, 16]. However, the underlying intestinal absorption mechanisms of monotropein and its ester derivatives remain poorly characterized.

In vitro absorption studies are frequently conducted using differentiated Caco‐2 cell monolayers, which mimic enterocytes with tight junctions and brush border enzymes. However, this model does not fully replicate intestinal physiology due to the absence of a mucus layer, which plays a critical role in the absorption of dietary phytochemicals by acting as a diffusion barrier and potential concentrating matrix for polar compounds [17]. Co‐culture systems incorporating mucus‐producing HT29‐MTX‐E12 goblet cells alongside Caco‐2 cells have been developed to overcome this limitation, providing greater physiological relevance while preserving experimental comparability [17].

The present study was therefore designed to characterize the intestinal uptake and transepithelial transport of monotropein and its esters using a validated co‐culture model of Caco‐2 and HT29‐MTX‐E12 human intestinal cells. To provide a mechanistic framework for the observed absorption behavior, the physicochemical and predicted pharmacokinetic properties of all three compounds were also assessed using in silico tools. Additionally, passive permeability was evaluated by PAMPA, transporter involvement was investigated using selective pharmacological inhibitors, and early epithelial metabolism was assessed by untargeted HPLC‐MS and enzymatic hydrolysis.

## 2 Experimental Section

### 2.1 Chemicals

HPLC‐grade solvents, culture media, supplements, and most reference compounds were obtained from Carl Roth (Karlsruhe, Germany) or Sigma‐Aldrich (Taufkirchen, Germany). Bovine serum albumin (BSA, cat. no. 23209), paraformaldehyde (cat. no. J61984.AP), and bicinchoninic acid (BCA) protein assay kit (cat no. 23225) were from Thermo Fisher Scientific (Dreieich, Germany). Pharmacological inhibitors were from MedChemExpress (Sollentuna, Sweden): sotaglifozin (cat. no. HY‐15516), phloretin (cat. no. HY‐N0142), AR‐C155858 (cat. no. HY‐13248), indinavir sulfate (cat. no. HY‐B0689A), fumitremorgin C (cat. no. HY‐N2143), PGP‐4008 (cat. no. HY‐119823), and MK‐571 (cat. no. HY‐19989). Monotropein was from BOC Sciences (Shirley, NY, USA; cat. no. B1370‐124565), and Lipoid E80 was from Lipoid (Ludwigshafen, Germany; cat. no. 510300).

### 2.2 Isolation of Monotropein Esters

Isolation of monotropein‐10‐*trans*‐coumarate and monotropein‐10‐*trans*‐cinnamate from *G. phillyreifolia* and *G. poeppigii* berries followed our previous procedure [6]. Briefly, MeOH–formic acid–H_2_O (70:1:29, *v*/*v*/*v*) extracts were fractionated using a Sartobind strong cation capsule. The co‐pigment fraction was submitted to counter‐current chromatography (CCC) using a QuikPrep MK8 Quattro instrument (AECS, Cornwall, UK). Separations were performed with coils 2+3 (total volume of 236 mL). After testing different biphasic polar solvent systems, *tert*‐butylmethylether/1‐butanol/acetonitrile/H_2_O (6:1:2:10, *v*/*v*/*v*/*v*), acidified with 0.1% trifluoroacetic acid was applied in a head‐to‐tail mode. The organic stationary phase was pumped into the coils 2+3 at 10 mL/min with an HPLC‐pump (Beta 50 Plus Gradient Pump, Ecom, Praha, Czech Republic). Once the coils were filled, rotation was set at 860 rpm. The aqueous mobile phase was then pumped at 2 mL/min. Once the hydrodynamic equilibrium between the phases was reached (stationary phase retention of 70.3%), the co‐pigment sample (500 mg) was dissolved in a mixture 1:1 (*v*/*v*) of the biphasic system, filtered through a 0.45 µm PTFE disk filter and injected into the CCC system from a 10 mL external loop (IDEX, Middleboro, MA, USA). A Flash 10 diode array detector (Ecom) was used to monitor compound elution at 280 nm for monotropein‐10‐*trans*‐cinnamate and 310 nm for monotropein‐10‐*trans*‐coumarate. After 40 min of injection, fractions of 4 mL were collected using a Gilson FC 203B fraction collector (Middleton, WI, USA). The solvent from the pooled fractions was evaporated in a rotary evaporator and the residual water was removed by freeze‐drying. The resulting sample was stored at −20°C until analysis by HPLC, as described in the following sections.

### 2.3 Cell Culture

Caco‐2 (ECACC 09042001) and HT29‐MTX‐E12 (ECACC 12040401) cells were obtained from the European Collection of Authenticated Cell Cultures (ECACC, Salisbury, UK). Caco‐2 cells were cultured in high‐glucose DMEM (4500 mg/L; cat. no. D5796, Sigma‐Aldrich) supplemented with fetal bovine serum (10%; cat. no. S0615, Sigma‐Aldrich), sodium pyruvate (1%; cat no. 9182.1, Carl Roth), non‐essential amino acids (1%, cat no. 9185.1, Carl Roth), and antibiotics (penicillin/streptomycin, 1%; cat no. 3A7X.1, Carl Roth). HT29‐MTX‐E12 cells were grown in supplemented high‐glucose DMEM but replacing sodium pyruvate with l‐glutamine (1%, cat. no. 9182.1, Carl Roth). Co‐culture medium consisted of high‐glucose DMEM supplemented with FBS (10%), non‐essential amino acids (1%), sodium pyruvate (1%), l‐glutamine (1%), and antibiotics (1%). Cultures were maintained at 37°C in a 5% CO_2_ humidified environment (HERAcell 150, Thermo Electron Corporation, Langenselbold, Germany). The culture medium was changed every 2–3 days.

### 2.4 Establishment and Validation of the Co‐Culture Model

Co‐cultures of Caco‐2 and HT29‐MTX‐E12 cells were carried out following the procedure described in the literature [18]. Briefly, cells were seeded in 12 mm insert polyester membrane Transwell permeable supports (1.2 cm^2^, 0.4 µm, Costar, Corning Inc., Kennebunk, ME, USA; cat. no. 3460) at a density of 5 × 10^5^ cells/insert. The ratios of Caco‐2:HT29‐MTX‐E12 cells that were evaluated were: 100:0, 0:100, 90:10, 75:25, and 50:50. After 21 days of incubation, cells from the apical side were washed with HBSS (pH 7.4) before the experiment.

Monolayer integrity was ensured by measuring the transepithelial electrical resistance (TEER) during the 21 days of differentiation using an EVOM‐G Ohm Meter (World Precision Instruments, Sarasota, FL, USA). TEER values were calculated according to Equation (1).

(1) $$\mathrm{TEE}R_{\mathrm{final}}\;\left(\Omega \;cm^{2}\right)=\left(\mathrm{TEE}R_{\mathrm{sample}}-\mathrm{TEE}R_{\mathrm{blank}}\right)\;(\Omega )\;x\;A\;(cm^{2})$$

where TEER_sample_ corresponds to the measured resistance of the inserts seeded with the co‐culture model, TEER_blank_ corresponds to the resistance of inserts without cells, and *A* is the area (1.2 cm^2^). Co‐culture inserts with TEER values > 300 Ω cm^2^ were used for subsequent experiments.

Lucifer yellow rejection assay was carried out to validate the integrity of the monolayer and the tight junctions of the co‐culture model. For this purpose, an aliquot of 100 µmol/L lucifer yellow (prepared in 1% DMSO in HBSS pH 6.0; cat no. L0144, Sigma‐Aldrich) was added to the apical side and cells were incubated for 3 h at 37°C, 5% CO_2_, and 50 rpm. At the end of the incubation time, the apical and basolateral concentration of lucifer yellow was determined by means of fluorescence detection (*λ*_ex/em_ 480/520 nm). The percentage of rejection was calculated according to Equation (2):

(2) $$\%\;\mathrm{Lucifer}\;\mathrm{yellow}\;\mathrm{rejection}=100\;\times \;(1-\left(\frac{LY_{\mathrm{basolateral}}}{LY_{\mathrm{apical}}}\right)$$

where LY_basolateral_ is the concentration of the dye in the basolateral chamber and LY_apical_ is the concentration of the dye in the apical chamber at the end of the incubation. Lucifer yellow rejection percentages above 99.5% indicate monolayer integrity [19]. The rationale for the final ratio selection based on these validation parameters is described in Section 3.3.

### 2.5 Visualization of Mucus by Alcian Blue Staining

**FIGURE 2:**
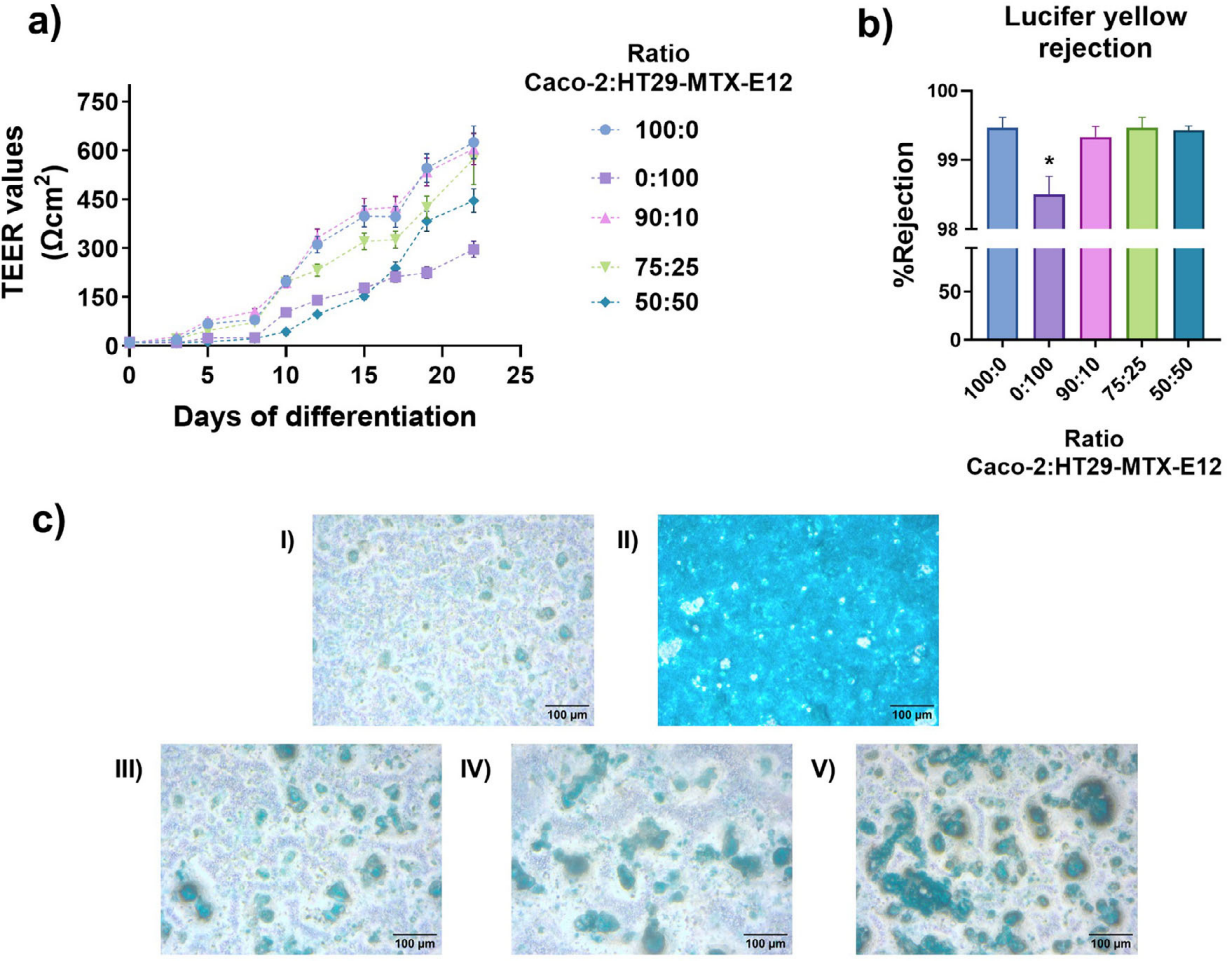
Validation of the Caco‐2:HT29‐MTX‐E12 co‐culture model. (a) Transepithelial electrical resistance (TEER) evolution in monocultures and co‐cultures (90:10, 75:25, and 50:50) over 21 days of differentiation. Data are means ± SD of two independent measurements per condition per timepoint. Statistical comparisons at day 22 were performed by two‐way ANOVA with Tukey's post hoc test. The 90:10 and 75:25 co‐cultures did not differ significantly from the Caco‐2 monoculture (100:0) (*p* = 0.9181 and *p* = 0.2357, respectively), while the HT29‐MTX‐E12 monoculture (0:100) showed significantly lower TEER than all other conditions (all *p* < 0.0001). The 50:50 co‐culture showed significantly lower TEER than both the Caco‐2 monoculture and the 90:10 and 75:25 co‐cultures (all *p* < 0.0001). (b) Lucifer yellow rejection (%) in differentiated monocultures and co‐cultures. Data are means ± SD of three independent biological replicates (*n* = 3). The 0:100 condition (HT29‐MTX‐E12 monoculture) showed significantly lower rejection than all other conditions (one‐way ANOVA with Tukey's post hoc test; 0:100 vs. 100:0: *p* = 0.0003; 0:100 vs. 90:10: *p* = 0.0009; 0:100 vs. 75:25: *p* = 0.0003; 0:100 vs. 50:50: *p* = 0.0004). All other pairwise comparisons were non‐significant (*p* > 0.86). (c) Alcian Blue 8GX staining of acidic mucins in (I) Caco‐2 monoculture, (II) HT29‐MTX‐E12 monoculture, and co‐cultures at ratios of (III) 90:10, (IV) 75:25 and (V) 50:50. Images were acquired using a Zeiss AxioCam ERc5s camera (ZEN 2012 software) at 10× magnification. Scale bar = 100 µm.

Acidic mucins produced by HT29‐MTX‐E12 cells were visualized by Alcian Blue 8GX staining [18]. Fixation of the cell monolayer was carried out using 4% paraformaldehyde and stained with 1% (*w*/*v*) Alcian Blue 8GX (cat. no. A5268, Sigma‐Aldrich) in 3% (*v*/*v*) acetic acid. Stained mucins were observed using an inverted microscope equipped with an AxioCam ERc5s camera (Zeiss, Jena, Germany) at 10× magnification. Images were acquired and processed using ZEN 2012 software (blue edition, v1.1.2.0, Zeiss). Results of the Alcian Blue staining across all co‐culture ratios are shown in Figure 2c and discussed in Section 3.3.

### 2.6 Dosage Information and Cell Viability

As monotropein and its esters remain stable and water‐soluble following gastrointestinal digestion [6], non‐digested compounds were used to assess intestinal transport and absorption mechanisms. Digestate cytotoxicity (Figure S1) precluded its use. Compounds (15 mmol/L) and transport inhibitors (prepared in water‐free DMSO) were aliquoted and stored at −80°C until use. On experimental days, aliquots were thawed at 37°C and compound integrity was verified by HPLC‐DAD.

Cell viability was assessed using the MTT assay after 24 h exposure to the different test compounds (1–100 µmol/L). At the end of the incubation, formazan crystals were dissolved in DMSO, and absorbance was measured at 580 nm (reference 660 nm). Results were expressed relative to untreated controls, and Triton X‐100 (0.1%; cat. no. T8787, Sigma‐Aldrich) served as a positive control.

### 2.7 Cellular Uptake of Monotropein and Monotropein Esters

Caco‐2 and HT29‐MTX‐E12 cells (90:10) were seeded in 12‐well plates (1 × 10^5^ cells/well) and differentiated for 21 days. Cells were pre‐incubated with 0.05% BSA in 10 mmol/L MES in HBSS buffer (pH 6.0) for 15 min at 5% CO_2_, 37°C, and 50 rpm. Test compounds (100 µmol/L each) were freshly prepared in the same buffer and added to the wells. Uptake was measured at different time points (0–60 min). At each timepoint, three technical replicate wells per compound were pooled prior to cell extraction to maximize extraction efficiency and minimize analytical noise. For mechanistic assessment of the uptake and metabolism, four independent replicate plates were incubated with compounds (100 µmol/L) for 1 h at 37°C for full activity, or at 4°C to inhibit enzymatic metabolism and transporter activity.

At the end of the incubation, cells were washed with 0.9% NaCl to remove extracellular compound before extraction, ensuring that measured concentrations reflect intracellular accumulation only. Cells were then quenched with cold MeOH:H_2_O (1:1, *v*/*v*), scraped and extracted with ice‐cold CHCl_3_ [20]. Tubes were agitated for 20 min at 1600 rpm and 4°C (ThermoMixer C, Eppendorf, Wesseling‐Berzdorf, Germany), centrifuged for 5 min at 16 100× *g* at 4°C, and an aliquot of the polar phase was recovered. The non‐polar phase was discarded without disturbing the interphase.

The recovered polar aliquot was dried in an RVC‐2‐25 CD plus centrifugal evaporator (Martin Christ Gefriertrockungsanlagen GmbH, Osterode am Harz, Germany) to remove remaining CHCl_3_ and MeOH. The aqueous residue was completely removed by freeze‐drying the samples (Lyoquest‐85, Azbil Telstar Technologies S.L.U, Terrassa, Spain). The dried samples were stored at −80°C until HPLC analysis.

The interphase containing proteins [20] was washed with ice‐cold MeOH and then centrifuged for 10 min at 16 100× *g* at 4°C. Afterward, the solvent was removed, and the pellet was dried in the centrifugal evaporator. Proteins were resuspended in 2.5% SDS (*w*/*v*) and the total protein content was determined using the Pierce BCA protein assay kit.

Putative phase II conjugates were investigated by enzymatic hydrolysis with β‐glucuronidase from *Helix pomatia* type H‐1 (G0751, Sigma‐Aldrich). Briefly, freeze‐dried samples were resuspended in 100 mmol/L sodium acetate buffer (pH 5.0) and mixed with the enzyme prepared at a final concentration of 3000 U/mL of β‐glucuronidase and 100 U/mL of sulfatase. Samples were incubated for 1 h at 37°C in a ThermoMixer shaker at 600 rpm under light protection. Reactions were stopped on ice and metabolites extracted with EtOAc:MeOH (95:5, v/v), centrifuged and dried before HPLC‐DAD‐MS analysis.

### 2.8 Transepithelial Transport

Caco‐2 and HT29‐MTX‐E12 cells (90:10) were seeded in Transwell inserts (1.2 cm^2^, 0.4 µm pore size, 5 × 10^5^ cells/insert) and differentiated for 21 days. The transport experiments were carried out by adding test compounds (100 µmol/L in 1% DMSO in HBSS, pH 6.0 or pH 7.4), to the apical side (0.5 mL), with HBSS (1 mL, pH 7.4) in the basolateral chamber. Plates were incubated at 37°C, 5% CO_2_, and 50 rpm. Basolateral aliquots (0.3 mL) were collected at 60, 90, 120, and 150 min and replaced with fresh buffer. Monolayer integrity was confirmed by measuring TEER (>300 Ω cm^2^) and lucifer yellow rejection (>99.5%) [19].

Apparent permeability coefficient (*P*_app_) was calculated according to Equation (3):

(3) $$P_{\mathrm{app}}(\mathrm{nm}/s)=\frac{C_{f}\;\times \;V\;}{t\;\times \;C_{i}\;\times \;A}$$

where *C*_f_ is the final basolateral concentration (µmol/L), *V* is the volume of the basolateral chamber (mL), *t* is time (s), *C*_i_ is the initial apical concentration (µmol/L) and *A* is the membrane area (cm^2^) [21].

### 2.9 Effect of Transport Protein Inhibitors

Transporter involvement was evaluated using pharmacological inhibitors of sodium–glucose transport protein 1 (SGLT1), glucose transporter protein 2 (GLUT2), monocarboxylic acid transporter (MCT), and the organic anion transporter protein (OATP); as well as the efflux proteins: breast cancer resistance protein (BCRP), P‐glycoprotein (P‐gp), and the multidrug resistance‐associated protein (MRP). These target transporters were selected given the molecular structure of the molecules (Figure 1), as well as their involvement in absorption, distribution, and metabolism of xenobiotics [22]. For uptake assays, cells were pre‐incubated (15 min) and co‐incubated with 20 µmol/L sotagliflozin (inhibitor of SGLT1), 200 µmol/L phloretin (inhibitor of GLUT2), 2 µmol/L AR‐C155858 (inhibitor of MCT), or 25 µmol/L indinavir sulfate (inhibitor of OATP). For the transport assay, inhibitors included 20 µmol/L fumitremorgin C (inhibitor of BCRP), 20 µmol/L PGP‐4008 (inhibitor of P‐gp), or 100 µmol/L MK‐571 (inhibitor of MRP). Assays were conducted in triplicate across three independent cell passages.

### 2.10 Parallel Artificial Membrane Permeability Assay (PAMPA)

Passive permeability was assessed by PAMPA [23]. MultiScreen‐IP PAMPA assay plates (MAIPNTR10) and PTFE Acceptor Plates (MATRNPS50) were obtained from Sigma‐Aldrich Chemie (Taufkirchen, Germany). Briefly, donor plates were coated with 5 µL of a solution composed of Lipoid E80 (1.7% *w*/*v*) and cholesterol (2.1% *w*/*v*, cat. no. C8667, Sigma‐Aldrich) in dodecane. Immediately after application, test compounds (100 µmol/L in PBS + 5% DMSO, pH 6.0 or 7.4) and reference compounds (furosemide, cat. no. HY‐B0135, MedChemExpress; propranolol, cat. no. HY‐B0573, MedChemExpress; and testosterone, cat. no. T1500, Sigma‐Aldrich) were loaded into the donor wells (150 µL), while the acceptor plate received 300 µL of 5% DMSO in PBS (pH 7.4). The PAMPA sandwich was assembled and incubated for 16 h in a humidified chamber at room temperature. After incubation, compound concentration in the donor and acceptor wells was determined by HPLC analysis.

The permeability coefficient through the artificial membrane (log *P*_E_) was calculated according to Equation (4) [24]:

(4) $$\log\;P_{E}=\log\left\{C\;\times \;\ln\left(1-\frac{{\left[\mathrm{Drug}\right]}_{\mathrm{acceptor}}}{{\left[\mathrm{Drug}\right]}_{\mathrm{donor}}}\right)\right\}\;$$

where [Drug] is the concentration calculated in the acceptor and donor chambers (nmol/mL), respectively, and *C* corresponds to the parameter calculated in Equation (5):

(5) $$C=\left(\frac{V_{D}\;\times \;V_{A}}{\left(V_{D}+V_{A}\right)\;\times \;A\;\times \;T}\right)$$

where *V*_D_ is the volume of the donor chamber (150 µL), *V*_A_ is the volume of the acceptor chamber (300 µL), *A* corresponds to the area of the PTFE membrane (0.302 cm^2^), and *T* is the incubation time (57 600 s). Results are presented as mean values of the calculated log *P*_E_ ± standard deviation (dimensionless) calculated from a minimum of three independent PAMPA plates.

### 2.11 HPLC‐DAD and HPLC‐MS Analysis of Monotropein and Monotropein Esters

A Nexera XR HPLC system (Shimadzu, Kyoto, Japan) equipped with a DGU‐403 degassing unit, two LC‐40D XR solvent delivery modules, a SL‐40C XR autosampler, a CTO‐40C column oven, and an SPD‐M40 photodiode array detector was used.

For monotropein, separation was achieved on an InertSustain AQ‐C18 column (5 µm, 4.6 × 150 mm) (GLSciences, Tokyo, Japan, cat. no. 5020–89730) using 50 mmol/L NaH_2_PO_4_ (pH 2.5) and MeOH (98:2, *v*/*v*), isocratic elution (1 mL/min, 30°C), monitored at 238 nm. For esters, a Kinetex PFP column (250 × 4.6 mm, 5 µm, Phenomenex, Torrance, CA, USA, cat. no. 00G‐4602‐E0) and mixtures of H_2_O:ACN:formic acid in proportions of (A) 88.5:3:8.5 (*v*/*v*/*v*) and (B) 41.5:50:8.5 (*v*/*v*/*v*) were used, monitoring at 280 nm (cinnamate) and 310 nm (coumarate). Calibration curves of monotropein (0.25–64 nmol/mL, *r* ^2^ = 0.9999), monotropein‐10‐coumarate (0.25‐30 nmol/mL, *r* ^2^ = 0.9989) and monotropein‐10‐cinnamate (0.25–30 nmol/mL, *r* ^2^ = 0.9991) were used for quantification.

Non‐targeted analysis was performed on a Q‐Exactive Plus Mass Spectrometer (Thermo Fisher Scientific, Waltham, MA, USA) coupled to a UPLC system. Compounds were separated on an Acquity HSS T3 column (100 Å, 1.8 µm, 2.1 × 150 mm, Waters, Eschborn, Germany), using H_2_O and MeOH gradients (0.2% formic acid) at 40°C and 0.3 mL/min. Data were acquired in both polarities over the *m*/*z* range of 100–1400 and analyzed with XCalibur 4.0 software.

### 2.12 In Silico Metabolite Prediction

Putative phase I and II metabolites were predicted using the GLORYx platform through the NERDD interface [25]. SMILES structures of monotropein and esters were submitted and metabolites with prediction scores >0.3 were selected [26]. Predicted masses and fragments were screened in HPLC‐MS data using the extracted ion chromatogram function.

### 2.13 In Silico Physicochemical Characterization

Physicochemical and pharmacokinetic properties of monotropein, monotropein‐10‐*trans‐*coumarate and monotropein‐10‐*trans*‐cinnamate were predicted using complementary in silico tools. Molecular formula, molecular weight, hydrogen bond donors and acceptors, rotatable bonds, topological polar surface area (TPSA), lipophilicity (consensus Log *P_o_*_/_ *_w_* and iLOGP), and water solubility (log S, ESOL method) were retrieved from the SwissADME webtool [27] using stereochemistry‐explicit SMILES strings. Gastrointestinal absorption, P‐glycoprotein substrate prediction, and bioavailability score were also obtained from SwissADME. Acid dissociation constants (p*K*_a_) were calculated using MarvinSketch v.25.3.120 (ChemAxon, Budapest, Hungary) applying the manufacturer's default parameters. Predicted intestinal absorption (% absorbed) and Caco‐2 permeability (log *P*_app_, expressed as log (10^−^^6^ cm/s)) were obtained from the pkCSM platform [28].

### 2.14 Statistical Analyses

Results are presented as mean values ± SD from at least three independent biological experiments unless otherwise stated. Normality was assessed using the D'Agostino–Pearson test. Comparisons were performed using one‐way ANOVA with Dunnett's or Tukey's post hoc test, or two‐way ANOVA with Tukey's or Šídák's post hoc test, as appropriate (GraphPad Prism 10.2.3, GraphPad Software, La Jolla, CA, USA), with *p* < 0.05 considered significant. Dunnett's post hoc test was used when comparing multiple treatment groups against a single control condition. Tukey's post hoc test was used for all pairwise comparisons. Šídák's post hoc test was used for planned comparisons between specific condition pairs

## 3 Results

### 3.1 Physicochemical Characterization of Monotropein and Monotropein Esters

The computed physicochemical properties of monotropein and its esters are summarized in Table 1. All three compounds exhibited high polarity, as reflected by TPSA values of 186.37 Å^2^ (monotropein), 212.67 Å^2^ (monotropein‐10‐*trans*‐coumarate), and 192.44 Å^2^ (monotropein‐10‐*trans*‐cinnamate), all above the 140 Å^2^ associated with poor passive membrane permeability [29]. Esterification progressively increased lipophilicity, with consensus Log *P_o_*_/_ *_w_* values rising from −2.45 (monotropein) to −0.79 (monotropein‐10‐coumarate) and −0.34 (monotropein‐10‐cinnamate), though all three compounds remained overall hydrophilic. The strongest acidic p*K*_a_ ranged from 4.10 to 4.14 across the series, corresponding to the carboxylic acid moiety at C4. The strongest basic p*K*_a_ of −2.98 was found for all compounds. All three compounds violated Lipinski's rule of five, with monotropein showing two violations (*NorO > 10, NHorOH > 5*) and both esters showing three violations (MW > 500, *NorO > 10, NHorOH > 5*). In silico Caco‐2 permeability predictions were low for all three compounds (log *P*_app_ −0.500, −0.615, and −0.551 for monotropein, monotropein‐10‐coumarate, and monotropein‐10‐cinnamate, respectively).

**TABLE 1:** Predicted physicochemical properties of monotropein, monotropein‐10‐*trans*‐coumarate, and monotropein‐10‐*trans*‐cinnamate.

Parameter	Monotropein	Monotropein‐10‐*trans*‐coumarate	Monotropein‐10‐*trans*‐cinnamate
Molecular properties			
Molecular formula	C_16_H_22_O_11_	C_25_H_28_O_13_	C_25_H_28_O_12_
MW (g/mol)	390.34	536.48	520.48
H‐bond donors	7	7	6
H‐bond acceptors	11	13	12
Rotatable bonds	5	9	9
TPSA (Å^2^)	186.37	212.67	192.44
Lipophilicity and solubility			
Log *P_o_*_/_ *_w_* (iLOGP)	0.44	1.71	2.33
Consensus Log *P_o_*_/_ *_w_*	−2.45	−0.79	−0.34
Log S (ESOL)	0.05	−2.17	−2.29
Solubility (mg/mL)	4.36 × 10^2^	3.66	2.67
Ionization			
p*K*_a_ strongest acidic	4.14	4.10	4.10
p*K*_a_ strongest basic	−2.98	−2.98	−2.98
Predicted absorption			
GI absorption	Low	Low	Low
Intestinal absorption (%)	4.57	13.35	21.61
Caco‐2 permeability (log *P*_app_)	−0.500	−0.615	−0.551
P‐glycoprotein substrate	Yes	Yes	Yes
Drug‐likeness			
Lipinski violations	2	3	3
Bioavailability score	0.11	0.11	0.11

Abbreviations: Caco‐2 permeability, predicted apparent permeability coefficient in Caco‐2 cells (log *P*_app_, expressed as log 10^−^^6^ cm/s); Consensus Log *P*_o/w_, average of five log P prediction methods (iLOGP, XLOGP3, WLOGP, MLOGP, SILICOS‐IT); GI, gastrointestinal; iLOGP, iterative logarithm of the partition coefficient, calculated using a physics‐based method; Log *P*_o/w*_, octanol–water partition coefficient; Log S (ESOL), predicted aqueous solubility using the ESOL method; MW, molecular weight; P‐glycoprotein, efflux transporter encoded by the ABCB1 gene; p*K*_a_, acid dissociation constant; TPSA, topological polar surface area;

### 3.2 Isolation of Monotropein‐10‐coumarate and Monotropein‐10‐cinnamate by CCC

The co‐pigment fraction obtained from *Gaultheria* spp. after cation‐exchange chromatography (Section 2.2) was submitted to CCC to isolate monotropein‐10‐cinnamate and monotropein‐10‐coumarate. Fourteen biphasic solvent systems based on *tert*‐butyl methyl ether, acetonitrile, 1‐butanol, water, and trifluoroacetic acid were screened to achieve partition coefficients (*K*_D_) of 0.5–2.5, and separation factors (*α* = *K*_D1_/*K*_D2_, with *K*_D2_ > *K*_D1_) > 1.5 (Table S1) [30]. Increasing the proportions of 1‐butanol improved early elution of non‐target matrix components (peak 1 in Figure S2) and enhanced separation from the monotropein esters. Using the final system (*tert*‐butyl methyl ether/1‐butanol/acetonitrile/H_2_O, 6:1:2:10, *v*/*v*/*v*/*v*, acidified with 0.1% of trifluoroacetic acid), monotropein‐10‐coumarate eluted after 187 mL and monotropein‐10‐cinnamate after 262 mL (Figure S2). HPLC‐DAD (254 nm) analysis of pooled fractions rich in analytes indicated purities of 81.3% and 72.1%, respectively. Stock solutions (15 mmol/L, water) were aliquoted and stored at −80°C for subsequent experiments.

### 3.3 Validation of the Caco‐2:HT29‐MTX‐E12 Co‐Culture

Monolayer integrity was monitored during 21 days of differentiation using TEER measurements, lucifer yellow rejection, and Alcian Blue 8GX staining. The different ratios of Caco‐2:HT29‐MTX‐E12 used for the assay showed significant differences in all the evaluated parameters. TEER rose progressively in all conditions (Figure 2a). On day 22, Caco‐2 monocultures showed the highest TEER (625 ± 50 Ω cm^2^), whereas HT29‐MTX‐E12 monocultures reached only 297 ± 25 Ω cm^2^. Co‐cultures showed intermediate integrity, achieving TEER values of 605 ± 48, 575 ± 79, and 446 ± 36 Ω cm^2^ for the 90:10, 75:25, and 50:50 seeding ratios (Caco‐2:HT29‐MTX‐E12), respectively. Statistical comparison at day 22 confirmed that the 90:10 and 75:25 co‐cultures did not differ significantly from the Caco‐2 monoculture (*p* = 0.9181 and *p* = 0.2357, respectively), while the HT29‐MTX‐E12 monoculture showed significantly lower TEER than all other conditions (all *p* < 0.0001). The 50:50 co‐culture showed significantly lower TEER than the Caco‐2 monoculture and the 90:10 and 75:25 co‐cultures (all *p* < 0.0001; two‐way ANOVA with Tukey's post hoc test). Caco‐2 monocultures and all the co‐culture ratios exhibited lucifer yellow rejection percentages higher than 99.5%, while the HT29‐MTX‐E12 monoculture reached a rejection percentage of 98.5% (Figure 2b). Alcian Blue 8GX staining revealed minimum mucin in Caco‐2 monocultures, robust mucus in HT29‐MTX‐E12 monocultures, and heterogeneous mucus distribution in co‐cultures (Figure 2c). Based on the barrier function and mucus production, the 90:10 co‐culture was selected for all further work and used in the absorption experiments described in Sections 3.5–3.9.

### 3.4 Cell Viability

Cytotoxicity was evaluated by MTT after 24 h exposure to monotropein, monotropein‐10‐coumarate, and monotropein‐10‐cinnamate (1–100 µmol/L). None of the compounds reduced the viability of the differentiated co‐culture (Figure S1), confirming that cytotoxicity at the highest tested concentration (100 µmol/L) remained below the 10% threshold commonly accepted for absorption studies [19]. No dose‐dependent cytotoxic trend was observed across the concentration range tested for any compound, and the sigmoidal dose–response relationship required for IC_50_ determination was not present; therefore, IC_50_ values could not be calculated. Based on these results, a maximum concentration of 100 µmol/L was selected for the following experiments to ensure detectable intracellular levels without compromising cell viability.

### 3.5 Cellular Uptake Kinetics

Cells were exposed to 100 µmol/L of each compound for 5, 15, 30, 45 and 60 min. Uptake experiments were conducted in 12‐well plates, which precluded from direct TEER measurement; cell viability under these conditions was confirmed by the MTT assay as described in Section 3.4. At each time point, three technical replicate wells per compound were pooled prior to cell extraction. After incubation, cells were washed with 0.9% NaCl to remove extracellular compound before extraction.

**FIGURE 3:**
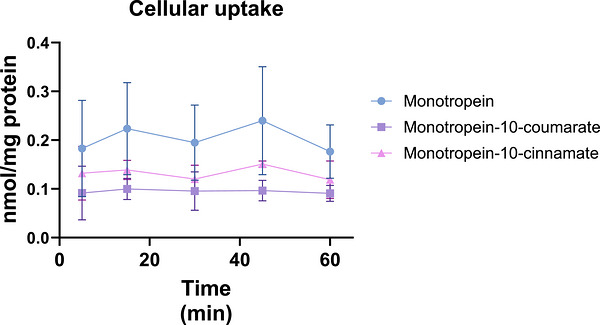
Time‐course cellular uptake of monotropein, monotropein‐10‐coumarate, and monotropein‐10‐cinnamate by the Caco‐2:HT29‐MTX‐E12 (90:10) co‐culture after incubation with 100 µmol/L of each compound at 37°C. Data are means ± SD of three independent biological replicates (*n* = 4 per time point). No statistically significant differences were observed between compounds at any time point (two‐way ANOVA with Tukey's post hoc test, *p* > 0.05 for all comparisons). Linear regression analysis confirmed no significant time‐dependent trend for any compound (monotropein: *r* ^2^ = 0.000, *p* = 0.973; monotropein‐10‐coumarate: *r* ^2^ = 0.074, *p* = 0.659; monotropein‐10‐cinnamate: *r* ^2^ = 0.036, *p* = 0.760).

Uptake was rapid and showed no clear time‐dependent accumulation plateau (Figure 3). At 5 min, intracellular levels (nmol/mg protein) were 0.183 ± 0.099 for monotropein, 0.092 ± 0.055 for monotropein‐10‐coumarate, and 0.132 ± 0.055 for monotropein‐10‐cinnamate. At 60 min, values were 0.176 ± 0.055, 0.091 ± 0.016, and 0.119 ± 0.038, respectively. Two‐way ANOVA with Tukey's post hoc test indicated no significant difference between the parent compound and either ester at any time point (*p* > 0.05), although monotropein‐10‐coumarate tended to be lower across all time points. Linear regression analysis confirmed the absence of any significant time‐dependent trend for all three compounds (monotropein: *r* ^2^ = 0.000, *p* = 0.973; monotropein‐10‐coumarate: *r* ^2^ = 0.074, *p* = 0.659; monotropein‐10‐cinnamate: *r* ^2^ = 0.036, *p* = 0.760). Overall, uptake appeared rapid and time‐independent under the conditions tested.

### 3.6 Effect of Apical pH on Transepithelial Flux

**FIGURE 4:**
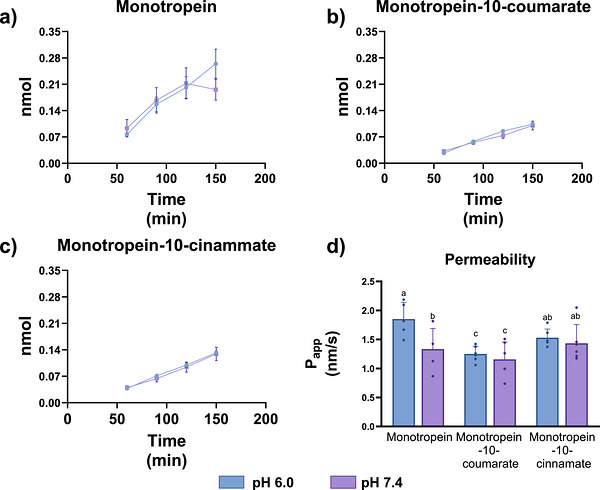
Effect of apical pH on transepithelial transport of (a) monotropein, (b) monotropein‐10‐coumarate, and (c) monotropein‐10‐cinnamate across Caco‐2:HT29‐MTX‐E12 (90:10) monolayers at pH 6.0 and pH 7.4; and (d) corresponding apparent permeability (*P*_app_, nm/s) after 150 min of incubation. Data are means ± SD of independent biological replicates (*n* = 5 for monotropein; *n* = 6 for monotropein‐10‐coumarate and monotropein‐10‐cinnamate). Different letters above bars in panel d indicate statistically significant differences (two‐way ANOVA with Tukey's post hoc test). Monotropein at pH 6.0 showed significantly higher *P*_app_ than monotropein at pH 7.4 (*p* = 0.0256), monotropein‐10‐coumarate at pH 6.0 (*p* = 0.0039), and monotropein‐10‐coumarate at pH 7.4 (*p* = 0.0009). All other pairwise comparisons were non‐significant (*p* ≥ 0.065).

Transport across the 90:10 co‐culture was assayed at apical pH 6.0 or 7.4 with sampling at 60, 90, 120, and 150 min. On all the experimental days, the TEER and lucifer yellow rejection (%) remained within the predefined acceptance limits (Table S2). For all three compounds, basolateral concentrations increased linearly over time (Figure 4). At 150 min, monotropein transport at pH 7.4 showed a lower but non‐significant value compared to transport observed at pH 6.0. The transport of monotropein was higher than that of the coumarate and cinnamate esters (Figure 4a–c). Apparent permeability coefficients (*P*_app_) after 150 min are shown in Figure 4d. Monotropein exhibited a significantly higher *P*_app_ at pH 6.0 than at pH 7.4 (1.851 ± 0.291 vs. 1.332 ± 0.357 nm/s, *p* = 0.0256). For monotropein‐10‐coumarate, *P*_app_ did not differ between pH 6.0 and 7.4 (1.251 ± 0.134 vs. 1.157 ± 0.298 nm/s, *p* > 0.05). Similarly, monotropein‐10‐cinnamate showed no pH effect (1.530 ± 0.148 vs. 1.434 ± 0.321 nm/s, *p* > 0.05). The *P*_app_ of monotropein exceeded that of monotropein‐10‐coumarate at both pH 6.0 (*p* = 0.0039) and pH 7.4 (*p* = 0.0009), while monotropein‐10‐cinnamate did not differ significantly from either of the other compounds (*p* > 0.065).

### 3.7 Temperature Dependence of Cellular Uptake

Cellular uptake was compared at 4°C versus 37°C over 1 h. The uptake experiments were conducted in 12‐well plates, which preclude direct TEER measurement; cell viability under these conditions was confirmed by the MTT assay as described in Section 3.4. Temperature had a significant effect on monotropein uptake, with intracellular concentrations significantly higher at 4°C than at 37°C (0.269 ± 0.026 vs. 0.141 ± 0.039 nmol/mg protein; two‐way ANOVA with Šídák's post hoc test, *p* < 0.0001). No significant temperature effect was observed for monotropein‐10‐coumarate or monotropein‐10‐cinnamate (both *p* > 0.9999, Figure S3). Protein content per well was slightly higher at 4°C than at 37°C across all conditions (2.34 ± 0.16 vs. 2.15 ± 0.15 mg/well; paired *t*‐test, *p* = 0.0049).

### 3.8 Metabolite Profiling

In silico predictions (Table S3) suggested phase II conjugation as a plausible metabolic pathway. For monotropein, *O*‐glucuronidation and *O*‐sulfation at the aliphatic carboxyl or hydroxyl groups were predicted. For monotropein‐10‐coumarate, *O*‐sulfation and *O*‐glucuronidation of the phenolic moiety, plus potential Michael‐type glucuronidation were predicted. For monotropein‐10‐cinnamate, only *O*‐glucuronidation at the aliphatic carboxyl was predicted. However, neither β‐glucuronidase/sulfatase treatment nor HPLC‐MS analysis of supernatants and cell pellets revealed glucuronides, sulfates, or other detectable metabolites after 1 h of incubation in the epithelial model. Free monotropein was not detected following co‐incubation with either ester.

### 3.9 Effect of Pharmacological Inhibitors of Transporters

**FIGURE 5:**
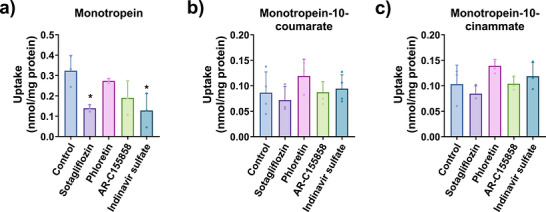
Effect of uptake transporter inhibitors on cellular accumulation of (a) monotropein, (b) monotropein‐10‐coumarate, and c) monotropein‐10‐cinnamate. Uptake was assessed after 60 min incubation (100 µmol/L, 37°C) with or without inhibitors of SGLT1 (sotagliflozin, 20 µmol/L), GLUT2 (phloretin, 200 µmol/L), MCT (AR‐C155858, 2 µmol/L), or OATP (indinavir sulfate, 25 µmol/L). Data are means ± SD of three independent biological replicates across three independent cell passages (*n* = 3). Statistical comparisons were performed by one‐way ANOVA with Dunnett's post hoc test using the control as reference. Monotropein uptake was significantly reduced by sotagliflozin (0.139 ± 0.016 nmol/mg protein; −57% vs. control, *p* = 0.0164) and indinavir sulfate (0.128 ± 0.083 nmol/mg protein; −60% vs. control, *p* = 0.0116). AR‐C155858 showed a non‐significant trend toward reduction (−41% vs. control, *p* = 0.0825). Phloretin had no effect (*p* = 0.7337). No significant differences were observed for monotropein‐10‐coumarate or monotropein‐10‐cinnamate with any inhibitor (all *p* > 0.05).

**FIGURE 6:**
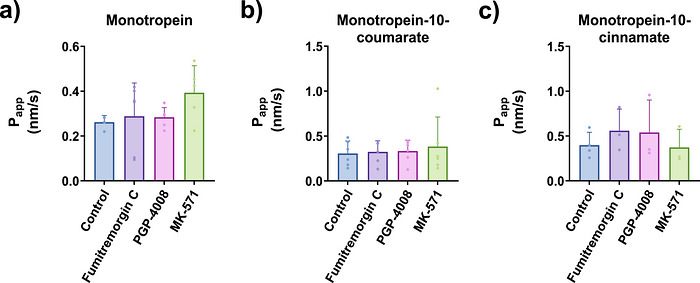
Effect of efflux transporter inhibitors on apparent permeability (*P*_app_, nm/s) of (a) monotropein, (b) monotropein‐10‐coumarate, and c) monotropein‐10‐cinnamate across Caco‐2:HT29‐MTX‐E12 (90:10) monolayers. *P*_app_ was determined after 150 min incubation (100 µmol/L, 37°C) with or without inhibitors of BCRP (fumitremorgin C, 20 µmol/L), P‐gp (PGP‐4008, 20 µmol/L), or MRP (MK‐571, 100 µmol/L). Data are means ± SD of three independent biological replicates across three independent cell passages (*n* = 3). No statistically significant differences were observed between any inhibitor condition and control for any of the three compounds (one‐way ANOVA with Dunnett's post hoc test, all *p* > 0.05), indicating no detectable contribution of efflux transporters to the transepithelial transport of monotropein or its esters under the tested conditions.

Transporter involvement was assessed after 15 min of pre‐incubation with the inhibitors followed by co‐incubation in the uptake and transport experiments. It should be noted that the uptake experiments described in this section (Figure 5) assess cellular accumulation, defined as intracellular compound concentration measured in cell lysates after washing, and are therefore distinct from the transepithelial transport experiments measuring basolateral appearance (Figures 4 and 6). Barrier integrity remained acceptable throughout the experiments (Table S4). Monotropein uptake (0.323 ± 0.075 nmol/mg protein) was significantly reduced by the SGLT1 inhibitor sotagliflozin (0.139 ± 0.016 nmol/mg protein; −57% relative to control, *p* = 0.0164) and the OATP inhibitor indinavir sulfate (0.128 ± 0.083 nmol/mg protein; −60% relative to control, *p* = 0.0116) (Figure 5a). Phloretin (inhibitor of GLUT2) and AR‐C155858 (inhibitor of MCT) produced modest, albeit non‐significant decreases (0.273 ± 0.011 and 0.190 ± 0.083 nmol/mg protein, respectively; −5% and −41% relative to control; both *p* > 0.05). In contrast, uptake of both esters was unaffected by any inhibitor (Figure 5b,c). For transepithelial transport, fumitremorgin C (inhibitor of BCRP), PGP‐4008 (inhibitor of P‐gp), and MK‐571 (inhibitor of MRP) did not significantly alter the *P*_app_ of monotropein or either ester (Figure 6).

### 3.10 Passive Diffusion Assessed by PAMPA

Passive permeability was examined by PAMPA using either a symmetric pH (pH apical → basolateral, 7.4 → 7.4) or pH gradient (pH 6.0 → 7.4). Plate integrity was confirmed with control compounds with known low permeability (furosemide), intermediate permeability (testosterone) and high permeability (propranolol), which yielded log *P*_E_ values in line with literature [24]. Furosemide presented a log *P*_E_ value of −8.17 ± 0.32, testosterone a log *P*_E_ value of −7.03 ± 0.03, and propranolol a log *P*_E_ value of −5.08 ± 0.04. Under these conditions, only monotropein‐10‐coumarate crossed the artificial membrane with log *P*_E_ value of −8.24 ± 0.14 at 6.0 → 7.4, and −8.18 ± 0.17 at pH 7.4 → 7.4. Monotropein and monotropein‐10‐cinnamate were not detected in the acceptor chamber after overnight incubation. Overall, the compounds exhibited low passive permeability in PAMPA, with detectable transfer only for monotropein‐10‐coumarate under both pH conditions tested.

## 4 Discussion

The intestinal epithelium is the primary site for dietary phytochemical absorption, functioning as a selective barrier and a site of pre‐systemic metabolism [31]. In vitro absorption studies are frequently conducted using differentiated Caco‐2 cell monolayers, which mimic enterocytes with tight junctions, limited paracellular diffusion, and brush border enzymes [32]. Although permeability data generated with this model correlate well with in vivo human studies [33], the Caco‐2 monolayer does not fully replicate intestinal physiology due to absence of mucus, fluid dynamics, cytochrome P450 expression, and differences in transporter profiles [17]. To overcome these limitations, co‐culture systems with mucus‐producing HT29‐MTX or its subclone E12 have been developed, adding complexity and physiological relevance, while preserving experimental comparability [34]. In this study, the 90:10 Caco‐2:HT29‐MTX‐E12 ratio was selected based on systematic evaluation of five seeding ratios. The higher TEER of the 90:10 co‐culture relative to the 50:50 ratio reflects the stronger barrier tightness conferred by the predominantly Caco‐2 composition, while the heterogeneous mucus distribution observed by Alcian Blue staining is consistent with the partial incorporation of mucus‐secreting goblet cells. Lucifer yellow rejection above 99.5% confirmed low paracellular permeability across all selected conditions. This selection is in agreement with previous studies using the same ratios [18, 35, 36] and highlights the importance of laboratory‐specific validation, as other studies have used ratios ranging from 1:1 to 70:30 [37, 38, 39, 40].

The absence of cytotoxic effects of monotropein and its esters across 1–100 µmol/L in the co‐culture model is consistent with earlier findings in Caco‐2 monolayers [5, 9], and with the reported non‐cytotoxicity of other iridoids including loganin (up to 80 µmol/L) [12], as well as morroniside and geniposide (up to 100 µmol/L) [13, 14]. The non‐cytotoxic profile of these compounds across multiple cell systems further supports the conclusion that the absence of cytotoxicity in the co‐culture model reflects a genuine property of these iridoids rather than a model‐specific artifact.

The physicochemical characterization of monotropein and its esters (Table 1) provides a mechanistic framework for interpreting their absorption behavior. All three compounds exhibited TPSA values above the 140 Å^2^ threshold associated with poor passive intestinal permeability [29], consistent with their predicted and observed low GI absorption. Despite esterification progressively increasing lipophilicity, with consensus Log *P_o_*_/_ *_w_* rising from −2.45 (monotropein) to −0.34 (monotropein‐10‐cinnamate), all three compounds remained overall hydrophilic and continued to violate multiple Lipinski rules, suggesting that molecular size rather than lipophilicity was the dominant limiting factor for passive permeability. The strongest acidic p*K*_a_ of ∼4.10–4.14 across the series, attributable to the carboxylic acid moiety at C4, indicates that all three compounds are predominantly ionized under both apical (pH 6.0) and basolateral (pH 7.4) intestinal conditions, which further limits their passive transcellular diffusion. Notably, while all three compounds were predicted as P‐glycoprotein substrates by in silico tools [27], no experimental evidence of P‐gp‐mediated efflux was observed under the tested conditions, suggesting that either the affinity for P‐gp is insufficient at the concentrations used (100 µmol/L) or that competing uptake processes mask any efflux contribution. In silico Caco‐2 permeability predictions (log *P*_app_ −0.500 to −0.615, expressed as log (10^−6^ cm/s)) were in qualitative agreement with the experimentally determined *P*_app_ values (1.2–1.9 nm/s), collectively supporting the conclusion that these iridoids face substantial physicochemical barriers to intestinal absorption.

Iridoids have poor to moderate oral absorption, largely due to their glycosylation and high polarity, which directly affect their bioavailability and metabolic fate [2]. In the literature, in situ intestinal perfusion in rats indicated that loganin uptake follows first‐order kinetics, consistent with passive diffusion [41]. While the time‐independent uptake profile observed for all three compounds (Section 3.5) is consistent with rapid passive equilibration, incubation at 4°C produced an unexpected increase in monotropein uptake (*p* < 0.0001) that is inconsistent with simple active transport inhibition at low temperature. This observation may instead reflect reduced efflux activity, inhibition of intracellular metabolism, or altered membrane partitioning at lower temperatures, any of which could increase net intracellular accumulation independent of active uptake. The slightly higher protein content at 4°C compared to 37°C (2.34 ± 0.16 vs. 2.15 ± 0.15 mg/well, *p* = 0.0049) is consistent with reduced proteolytic enzyme activity rather than cell death, confirming that cells remained intact and metabolically viable throughout the 4°C incubation [42]. For the esterified forms, no significant temperature effect was observed (both *p* > 0.9999), supporting passive diffusion as the predominant uptake mechanism. Together with the transporter inhibitor data (Section 3.9), these findings suggest a mixed mechanism for monotropein involving passive diffusion with partial transporter‐mediated contribution, rather than predominantly active uptake.

The gastrointestinal pH plays a decisive role in the absorption of iridoids, as demonstrated by the impact of the pH on the *P*_app_ of loganin and morroniside [12, 13]. The significantly higher *P*_app_ of monotropein at pH 6.0 versus 7.4 is consistent with the increase in non‐ionized fraction predicted by the Henderson–Hasselbalch equation [43]. Using the strongest acidic p*K*_a_ of 4.14 (Table 1), the predicted neutral fraction increases approximately 25‐fold from pH 7.4 to pH 6.0 (0.055% vs. 1.37%), directly supporting the observed pH‐dependent permeability. Ionization status is a well‐recognized determinant of membrane permeability [7], and the present findings are also consistent with previous reports of pH‐dependent monotropein absorption, although earlier studies attributed this effect to compound stability rather than ionization, given reports of spontaneous conversion to deacetylasperulosidic acid under acidic conditions [44], a reaction not observed in other gastrointestinal digestion models [5, 16]. For the esterified forms, the identical p*K*_a_ across the series (∼4.10, Table 1) yet absence of pH‐dependent permeability confirms that molecular size rather than ionization state was the dominant barrier to absorption of the esterified derivatives.

The absence of detectable phase I or II metabolites after 1 h of incubation in the epithelial model contrasts with in silico predictions by GLORYx, which suggested sulfatation, glucuronidation, and glutathione conjugation as plausible pathways, in line with in vivo data for other iridoids [45, 46]. The 1‐h incubation windows was selected to maintain consistency with the uptake experiments and to minimize compound degradation; however, phase II conjugations reactions such as glucuronidation and sulfation may require longer incubation times to produce detectable metabolite levels. The absence of metabolites is therefore most likely attributable to the inherently low metabolic capacity of Caco‐2 and HT29‐MTX‐E12 cells, particularly their low CYP450 expression [47, 48] and limited phase II conjugation activity, rather than reflecting a genuine absence of metabolic potential in vivo. In general, iridoid glycosides undergo aglycone formation followed by phase I and II modifications in vivo [2], and future studies using longer incubation times or metabolically competent cell systems, such as HepaRG or primary enterocytes would be needed to fully characterize epithelial metabolism of these compounds. Colonic microbial and hepatic metabolism are also known to extensively modify iridoids [49] and represent an important complementary routes for future investigation. Finally, it should be noted that the GLORYx model does not account for tridimensional substrate‐enzyme configurations and does not include human carboxylesterase 2 [26], the enzyme responsible for ester hydrolysis that could be relevant for monotropein esters in vivo.

**FIGURE 7:**
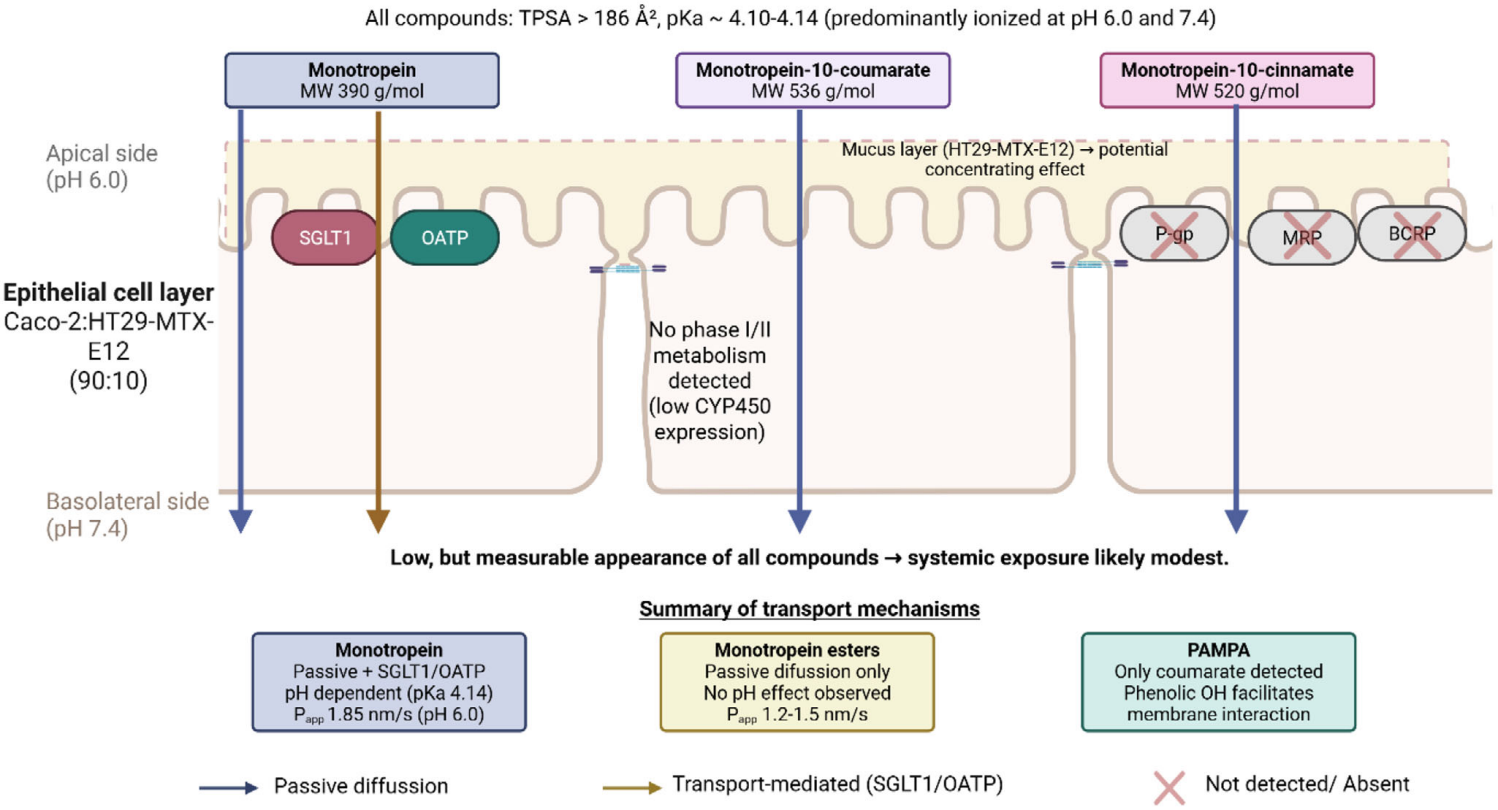
Proposed intestinal absorption mechanisms of monotropein, monotropein‐10‐coumarate, and monotropein‐10‐cinnamate in the Caco‐2:HT29‐MTX‐E12 (90:10) co‐culture model. All three compounds exhibit high polarity (TPSA > 186 Å^2^) and are predominantly ionized at intestinal pH (p*K*_a_ ∼ 4.10–4.14), consistent with their limited passive permeability. Monotropein uptake involves both passive diffusion and partial transporter‐mediated uptake via SGLT1 and OATP at the apical membrane, while esterified forms rely predominantly on passive diffusion. No detectable contribution of efflux transporters (P‐gp, BCRP, MRP) was observed for any compound. No phase I or II metabolites were detected under the tested conditions, consistent with the inherently low CYP450 expression and limited phase II conjugation capacity of the co‐culture model. Overall, basolateral appearance of all compounds was low, indicating that systemic exposure following dietary intake is likely modest. Created with BioRender (BioRender.com, Agreement number: FK29MGBZK3).

Partial involvement of SGLT1 and OATP in monotropein uptake, but not in that of its esters, was demonstrated by the inhibitor experiments (Section 3.9). These experiments assessed intracellular accumulation rather than transepithelial flux and should therefore be interpreted as reflecting apical membrane uptake into enterocytes rather than complete barrier crossing. The substantial reduction in monotorpein uptake by sotagliflozin and indinavir sulfate (−57% and −60%, respectively), indicates that transporter‐mediated uptake contributes meaningfully to cellular accumulation, consistent with the known capacity of glycosylated xenobiotics to act as SGLT1 substrates [50]. We have recently reported that monotropein and its esters can decrease glucose uptake in Caco‐2 cells [5], and the present findings are consistent with competitive antagonism at the SGLT1 transporter as a contributing mechanism. Intestinal OATP are increasingly recognized as mediators of phytochemical absorption and food‐drug interactions [14], and similar transporter mediated uptake has been described for the structurally related iridoid catalposide, where uptake was 319‐, 13.6‐ and 9.3‐fold greater in HEK293 cells overexpressing OAT3, OATP1B1, and OATP1B3, respectively, and was reversed by specific inhibitors [51]. Together with the PAMPA results (Section 3.10), these findings suggest that monotropein absorption combines passive diffusion with apical transporter‐mediated uptake, whereas the esterified forms rely predominantly on passive diffusion. The proposed absorption mechanisms are summarized schematically in Figure 7. No efflux transport was detected, in agreement with previous studies showing that iridoids such as geniposide are not P‐gp substrates [52]. This is noteworthy given the in silico prediction of P‐gp substrate activity for all three compounds [27], and may reflect concentration‐dependent transporter saturation at the concentration used (100 µmol/L) or overestimation of efflux liability by prediction tools for highly polar glycosylated compounds with multiple hydrogen bond donors, whose limited membrane partitioning may be insufficient to engage P‐gp despite predicted substrate recognition.

The discrepancy between the absence of detectable passive permeability in PAMPA and the measurable transepithelial transport observed in the co‐culture model warrants careful consideration. PAMPA is a purely passive, protein‐free artificial membrane system that exclusively captures transcellular diffusion driven by the physicochemical properties of the compound [23], whereas the Caco‐2:HT29‐MTX‐E12 co‐culture incorporates multiple transport mechanisms, a mucus layer, and membrane‐associated proteins that collectively facilitate compound movement. Several factors may explain this discrepancy. First, the partial SGLT1 and OATP involvement demonstrated in Section 3.9 provides a direct mechanistic basis for cellular transport in the absence of meaningful passive permeability, since transporter‐mediated uptake can sustain measurable intracellular accumulation and basolateral appearance even when passive diffusion is negligible [22]. Second, the mucus layer produced by HT29‐MTX‐E12 cells may act as a concentrating matrix for polar compounds through electrostatic interactions and hydrogen bonding, potentially facilitating their interaction with apical membrane transporters [34], an effect absent in PAMPA. Third, differences in unstirred water layer (UWL) thickness between the PAMPA sandwich and the Transwell system under orbital shaking (50 rpm) may reduce the diffusion resistance in the cell‐based system [23]. For monotropein‐10‐coumarate, detectable PAMPA transfer is consistent with its higher lipophilicity and the additional aromatic system relative to monotropein (Table 1). The absence of PAMPA signal for monotropein‐10‐cinnamate, despite its structural similarity to the coumarate ester, may relate to the lack of an ionizable phenolic hydroxyl group, which could reduce interaction with the phosphatidylcholine headgroups of the Lipoid E80 membrane. The similar *P*_app_ values for both esters in the cell model suggest that limited passive mechanisms are also operative, although the maintained lucifer yellow rejection >99.5% throughout the transporter experiments argues against a significant paracellular contribution.

In summary, monotropein and its naturally occurring ester derivatives exhibit limited intestinal permeability in the validated Caco‐2:HT29‐MTX‐E12 co‐culture model. The physicochemical characterization revealed that high polarity (TPSA > 186 Å^2^) and predominantly ionized states at intestinal pH are the primary barriers to passive absorption for all three compounds. Monotropein uptake involves a mixed mechanism combining passive diffusion with partial SGLT1‐ and OATP‐mediated uptake, is pH‐dependent consistent with its p*K*_a_ of 4.14, and shows a paradoxical increase at 4°C most likely reflecting reduced efflux or intracellular metabolic activity rather than active transport. Esterification progressively increased lipophilicity but did not enhance permeability, likely due to a molecular size penalty offsetting any lipophilicity gain, and the esters showed no transporter involvement or pH dependence. No efflux pump contribution, phase I/II epithelial metabolism, or ester hydrolysis was detected. The absence of detectable passive permeability in PAMPA for monotropein and monotropein‐10‐cinnamate, contrasting with measurable cell‐based transport, highlights the contribution of transporter‐mediated mechanisms and mucus layer effects not captured by cell‐free permeability assays. Collectively, this study provides the first mechanistic characterization of intestinal absorption for monotropein and its ester derivatives, establishing a physicochemical and transport basis for their limited systemic bioavailability. Future investigations should integrate colonic microbial metabolism, hepatic biotransformation, and metabolically competent cell systems to fully define the nutritional relevance of these dietary iridoids.

## Author Contributions

**Christian Zielinski**: conceptualization, formal analysis, investigation, methodology, validation, visualization, writing – review and editing. **Victor Schmalle**: formal analysis, investigation, methodology, writing – review and editing. **Luise A. Lauer**: formal analysis, investigation, methodology, writing – review and editing. **Tim Hammerschick**: investigation, methodology, writing – review and editing. **Felix Rüttler**: investigation, methodology, writing – review and editing. **Walter Vetter**: investigation, methodology, writing – review and editing. **Jan Frank**: funding, formal analysis, supervision, writing – review and editing. **Felipe Jiménez‐Aspee**: conceptualization, formal analysis, funding acquisition, investigation, methodology, project administration, supervision, validation, visualization, writing – original draft, writing – review and editing.

## Conflicts of Interest

The authors declare no conflicts of interest.

## Supporting information




**Supporting File**: mnfr70571‐sup‐0001‐SuppMat.docx.

## Data Availability

The data that support the findings of this study are available from the corresponding author upon reasonable request.
