# Supplementary material for: Uptake and Transport of Monotropein and Monotropein Esters in a Caco‐2/HT29‐MTX‐E12 Co‐Culture Model
Source: Mol Nutr Food Res. 2026 Jul 30;70(15):e70571. doi: 10.1002/mnfr.70571 (PMC13424965; doi:10.1002/mnfr.70571)
Supplement: Supplementary file 1 — Supporting File: mnfr70571‐sup‐0001‐SuppMat.docx. [file MNFR-70-e70571-s001.docx]

**Supplementary material**

**Uptake and transport of monotropein and monotropein esters in a Caco-2/HT29-MTX-E12 co-culture model**

Christian ZIELINSKI^1^, Victor SCHMALLE^1^, Luise A. LAUER^1^, Tim HAMMERSCHICK^2^, Felix RÜTTLER^2^, Walter VETTER^2^, Jan FRANK^1^, and Felipe JIMÉNEZ-ASPEE^1,*^

*^1^Department of Food Biofunctionality (140b), Institute of Nutritional Sciences, University of Hohenheim, 70599 Stuttgart, Germany.* E-mail addresses: [christian.zielinski@uni-hohenheim.de](mailto:christian.zielinski@uni-hohenheim.de) (C.Z); [v.schmalle@uni-hohenheim.de](mailto:v.schmalle@uni-hohenheim.de) (V.S.); luise.lauer@nutres.de (L.L); jan.frank@nutres.de (J.F); [felipe.jimenez@nutres.de](mailto:felipe.jimenez@nutres.de) (F.J.A.).

*^2^Institute of Food Chemistry (170b), University of Hohenheim, 70599 Stuttgart, Germany.* E-mail addresses: [tim.hammerschick@uni-hohenheim.de](mailto:tim.hammerschick@uni-hohenheim.de) (T.H.); [felix.ruettler@uni-hohenheim.de](mailto:felix.ruettler@uni-hohenheim.de) (F.R.); [walter.vetter@uni-hohenheim.de](mailto:walter.vetter@uni-hohenheim.de) (W.V.)

**Corresponding author: F. Jiménez-Aspee,* [*felipe.jimenez@nutres.de*](mailto:felipe.jimenez@nutres.de)

**Table S1.** Biphasic solvent composition, partition coefficients (K_D_) and separation factor (α) between both monotropein esters and the main impurities present in the copigment fraction.

| **System** | **Proportion**  ***tert*-butylmethyleter:1-butanol:Acetonitrile:H_2_O** | **Impurity**  **K_D_** | **Monotropein-10-coumarate**  **K_D_** | **Monotropein-10-cinnamate**  **K_D_** | **α** |
| --- | --- | --- | --- | --- | --- |
| 1 | 2:2:0:3 | 0.55 | 0.65 | 1.32 | 2.0 |
| 2 | 4:5:0:3 | 1.49 | 1.6 | 2.96 | 1.8 |
| 3 | 2:3:0:3 | 2.13 | 2.23 | 3.98 | 1.8 |
| 4 | 3:3:0:3 | 0.63 | 0.65 | 1.24 | 1.9 |
| 5 | 6:7:0:6 | 0.79 | 0.82 | 1.54 | 1.9 |
| 6 | 3:4:0:3 | 0.3 | 0.37 | 0.57 | 1.5 |
| 7 | 4:2:0:3 | 0.44 | 0.50 | 1.31 | 2.6 |
| 8 | 4:3:0:3 | 0.56 | 0.57 | 1.24 | 2.1 |
| 9 | 4:4:0:3 | 0.54 | 0.56 | 1.12 | 2.2 |
| 10 | 3:1:1:5 | 1.13 | 2.0 | 3.76 | 1.9 |
| 11 | 5:2:4:10 | 5.99 | 7.6 | 14.0 | 1.9 |
| 12 | 3:1:2:5 | 5.87 | 9.04 | 16.5 | 1.8 |
| 13 | 12:4:1:20 | 1.24 | 1.93 | 4.62 | 2.4 |
| 14 | 6:2:1:10 | 0.25 | 0.71 | 1.16 | 1.6 |

**Table S2.** Validation of the cell monolayer integrity in the transport assay of monotropein and monotropein esters.

|  | **pH apical** | **Replica** | **TEER value**  **(Ωcm^2^)** | | **% Lucifer yellow rejection** |
| --- | --- | --- | --- | --- | --- |
|  |  |  | **Initial** | **Final** |  |
| **Experimental day 1** | | | | | |
| Monotropein | 6.0 | 1 | 550 | 515 | 99.5 |
|  |  | 2 | 542 | 525 | 99.8 |
|  |  | 3 | 550 | 550 | 99.7 |
|  | 7.4 | 1 | 548 | 490 | 99.4 |
|  |  | 2 | 564 | 530 | 99.9 |
|  |  | 3 | 583 | 520 | 99.8 |
| Monotropein-10-coumarate | 6.0 | 1 | 565 | 562 | 99.8 |
|  |  | 2 | 520 | 550 | 99.8 |
|  |  | 3 | 545 | 580 | 99.7 |
|  | 7.4 | 1 | 550 | 560 | 99.9 |
|  |  | 2 | 550 | 530 | 99.8 |
|  |  | 3 | 589 | 600 | 99.8 |
| Monotropein-10-cinnamate | 6.0 | 1 | 566 | 690 | 99.9 |
|  |  | 2 | 593 | 588 | 99.8 |
|  |  | 3 | 560 | 608 | 99.8 |
|  | 7.4 | 1 | 593 | 580 | 99.8 |
|  |  | 2 | 589 | 550 | 99.8 |
|  |  | 3 | 570 | 600 | 99.4 |
| **Experimental day 2** | | | | | |
| Monotropein | 6.0 | 1 | 580 | 475 | 99.5 |
|  |  | 2 | 550 | 780 | 99.8 |
|  |  | 3 | 624 | 510 | 99.7 |
|  | 7.4 | 1 | 540 | 550 | 99.4 |
|  |  | 2 | 530 | 470 | 99.9 |
|  |  | 3 | 570 | 600 | 99.7 |
| Monotropein-10-coumarate | 6.0 | 1 | 570 | 520 | 99.8 |
|  |  | 2 | 600 | 500 | 99.9 |
|  |  | 3 | 610 | 540 | 99.7 |
|  | 7.4 | 1 | 590 | 620 | 99.9 |
|  |  | 2 | 590 | 660 | 99.8 |
|  |  | 3 | 600 | 680 | 99.9 |
| Monotropein-10-cinnamate | 6.0 | 1 | 570 | 550 | 99.9 |
|  |  | 2 | 615 | 550 | 99.7 |
|  |  | 3 | 590 | 570 | 99.7 |
|  | 7.4 | 1 | 540 | 630 | 99.8 |
|  |  | 2 | 600 | 630 | 99.4 |
|  |  | 3 | 590 | 650 | 99.8 |

**Table S3.** Predicted GLORYx phase I and II biotransformations of monotropein, monotropein-10-coumate and monotropein-10-cinnamate.

| **Parent compound** | **Score** | **Biotransformation** | **Predicted Structure** | **Molecular formula** | **Molecular weight (g/mol)** |
| --- | --- | --- | --- | --- | --- |
| Monotropein | 0.51 | O-glucuronidation aliphatic carboxyl |  | C_22_H_30_O_17_ | 566.46 |
|  | 0.41 | O-glucuronidation aliphatic hydroxyl |  | C_22_H_30_O_17_ | 566.46 |
|  | 0.32 | Sulfation, aliphatic hydroxyl |  | C_16_H_22_O_14_S | 470.40 |
| Monotropein-10-coumarate | 0.96 | Sulfation, aromatic hydroxyl aromatic |  | C_25_H_28_O_16_S | 616.54 |
|  | 0.94 | O-glucuronidation aromatic hydroxyl |  | C_31_H_36_O_19_ | 712.61 |
|  | 0.34 | Glutathione conjugation (alpha-beta-unsaturated carbonyl) |  | C_35_H_45_N_3_O_19_S | 843.81 |
|  | 0.34 | O-glucuronidation (aliphatic carboxyl) |  | C_31_H_36_O_19_ | 712.61 |
| Monotropein-10-cinnamate | 0.37 | O-glucuronidation aliphatic carboxyl |  | C_31_H_36_O_18_ | 696.61 |
|  | 0.31 | Glutathione conjugation (alpha,beta-unsaturated carbonyl) |  | C_35_H_45_O_18_S | 827.81 |

**Table S4**. Validation of the cell monolayer integrity in the transport assay in the presence of different pharmacological inhibitors.

|  | **Inhibitor** | **Replica** | **TEER value**  **(Ωcm^2^)** | | **% Lucifer yellow rejection** |
| --- | --- | --- | --- | --- | --- |
|  |  |  | **Initial** | **Final** |  |
| **Experimental day 1** | | | | | |
| Monotropein | Control | 1 | 1220 | 1600 | 99.9 |
|  |  | 2 | 1250 | 1520 | 99.9 |
|  |  | 3 | 1260 | 1620 | 99.9 |
|  | Fumitremorgin | 1 | 1200 | 1460 | 99.9 |
|  |  | 2 | 1380 | 1570 | 99.9 |
|  |  | 3 | 1330 | 1620 | 99.9 |
|  | PGP-4008 | 1 | 1220 | 1520 | 99.9 |
|  |  | 2 | 1300 | 1660 | 99.9 |
|  |  | 3 | 1400 | 1560 | 99.9 |
|  | MK-571 | 1 | 1200 | 1400 | 99.9 |
|  |  | 2 | 1230 | 1700 | 99.9 |
|  |  | 3 | 1230 | 1650 | 99.9 |
| Monotropein-10-coumarate | Control | 1 | 1200 | 1760 | 99.9 |
|  |  | 2 | 1260 | 1750 | 99.9 |
|  |  | 3 | 1250 | 1850 | 99.9 |
|  | Fumitremorgin | 1 | 1250 | 1620 | 99.9 |
|  |  | 2 | 1410 | 1900 | 99.9 |
|  |  | 3 | 1370 | 1750 | 99.9 |
|  | PGP-4008 | 1 | 1320 | 1820 | 99.9 |
|  |  | 2 | 1270 | 1730 | 99.9 |
|  |  | 3 | 1410 | 1850 | 99.9 |
|  | MK-571 | 1 | 1220 | 1960 | 99.9 |
|  |  | 2 | 1210 | 2000 | 99.9 |
|  |  | 3 | 1320 | 2050 | 99.9 |
| Monotropein-10-cinnamate | Control | 1 | 1440 | 1425 | 99.9 |
|  |  | 2 | 1150 | 1788 | 99.9 |
|  |  | 3 | 1220 | 1820 | 99.9 |
|  | Fumitremorgin | 1 | 1310 | 1900 | 99.9 |
|  |  | 2 | 1310 | 1900 | 99.9 |
|  |  | 3 | 1300 | 1930 | 99.7 |
|  | PGP-4008 | 1 | 1270 | 1825 | 99.9 |
|  |  | 2 | 1240 | 1830 | 99.9 |
|  |  | 3 | 1300 | 1970 | 99.9 |
|  | MK-571 | 1 | 1180 | 1760 | 99.9 |
|  |  | 2 | 1280 | 1930 | 99.9 |
|  |  | 3 | 1330 | 2070 | 99.9 |
| **Experimental day 2** | | | | | |
| Monotropein | Control | 1 | 1077 | 1490 | 99.9 |
|  |  | 2 | 1120 | 1540 | 99.9 |
|  |  | 3 | 1120 | 1520 | 99.9 |
|  | Fumitremorgin | 1 | 1120 | 1420 | 99.9 |
|  |  | 2 | 1060 | 1450 | 99.9 |
|  |  | 3 | 1100 | 1430 | 99.9 |
|  | PGP-4008 | 1 | 1100 | 1460 | 99.9 |
|  |  | 2 | 1050 | 1250 | 99.9 |
|  |  | 3 | 1150 | 1420 | 99.9 |
|  | MK-571 | 1 | 1120 | 1100 | 99.9 |
|  |  | 2 | 1150 | 1130 | 99.9 |
|  |  | 3 | 1150 | 1150 | 98.0* |
| Monotropein-10-coumarate | Control | 1 | 1120 | 1630 | 99.9 |
|  |  | 2 | 1130 | 1640 | 99.9 |
|  |  | 3 | 1100 | 1580 | 99.9 |
|  | Fumitremorgin | 1 | 1100 | 1600 | 99.9 |
|  |  | 2 | 1067 | 1500 | 99.9 |
|  |  | 3 | 1040 | 1400 | 99.9 |
|  | PGP-4008 | 1 | 1120 | 1570 | 99.9 |
|  |  | 2 | 1100 | 1600 | 99.9 |
|  |  | 3 | 1127 | 1520 | 99.9 |
|  | MK-571 | 1 | 1140 | 1680 | 99.9 |
|  |  | 2 | 1150 | 1680 | 99.9 |
|  |  | 3 | 1160 | 1760 | 99.9 |
| Monotropein-10-cinnamate | Control | 1 | 1092 | 1700 | 99.9 |
|  |  | 2 | 1100 | 1700 | 99.9 |
|  |  | 3 | 1140 | 1690 | 99.9 |
|  | Fumitremorgin | 1 | 1090 | 1620 | 99.9 |
|  |  | 2 | 1050 | 1660 | 99.9 |
|  |  | 3 | 1024 | 1700 | 99.7 |
|  | PGP-4008 | 1 | 1140 | 1670 | 99.9 |
|  |  | 2 | 1100 | 1750 | 99.9 |
|  |  | 3 | 1140 | 1950 | 99.9 |
|  | MK-571 | 1 | 1150 | 1940 | 99.9 |
|  |  | 2 | 1150 | 1920 | 99.9 |
|  |  | 3 | 1130 | 1150 | 99.9 |

* data from this well was not included in the analysis due to the low %lucifer yellow rejection.

**Table S5.** Cell viability (% of basal control) of monotropein, monotropein-10-coumarate and monotropein-10-cinnamate in the Caco-2:HT29-MTX-E12 co-culture model assessed by MTT assay after 24 h exposure. Data from three independent biological experiments performed on separate cell passages are shown individually together with the mean ± SD. Triton X-100 (0.1% v/v) was included as a positive control for cell death.

| **Compound** | **Concentration** | **P1** | **P2** | **P3** | **Mean ± SD** |
| --- | --- | --- | --- | --- | --- |
| Basal control | - | 100 | 100 | 100 | 100 ± 0 |
| Triton X-100 | - 1. %, v/v | 2.16 | 3.50 | 3.53 | 3.1 ± 0.8 |
| Monotropein | 1 µmol/L | 99.7 | 117.3 | 103.8 | 106.9 ± 9.0 |
|  | 10 µmol/L | 82.7 | 119.0 | 89.8 | 97.2 ± 19.4 |
|  | 100 µmol/L | 82.4 | 114.5 | 113.9 | 103.6 ± 18.3 |
| Monotropein-10-coumarate | 1 µmol/L | 102.2 | 109.5 | 93.2 | 101.7 ± 8.2 |
|  | 10 µmol/L | 73.2 | 101.8 | 101.7 | 92.2 ± 16.5 |
|  | 100 µmol/L | 85.4 | 130.5 | 119.6 | 111.8 ± 23.5 |
| Monotropein-10-cinnamate | 1 µmol/L | 107.4 | 130.7 | 85.2 | 107.8 ± 22.8 |
|  | 10 µmol/L | 80.6 | 104.1 | 93.2 | 92.7 ± 11.8 |
|  | 100 µmol/L | 93.4 | 142.5 | 126.7 | 120.9 ± 25.0 |

P = passage. Values are normalized to the basal control within each independent experiment; therefore the basal control mean is 100% by definition

**Figure S1.** Cytotoxicity of monotropein, monotropein-10-coumarate and monotropein-10-cinnamate in the Caco-2:HT29-MTX-E12 co-culture model as evaluated by the MTT assay after 24 h exposure. Data are means ± SD of three independent biological replicates (n = 3 per concentration). Cell viability remained above 90% of the untreated control at all tested concentrations (1–100 µmol/L), confirming the absence of cytotoxic effects at the concentrations used in subsequent experiments. No IC_50_ value could be determined as no sigmoidal dose-response relationship was observed. Triton X-100 (0.1% v/v) was included as a positive control for cell death. Asterisk (*) indicates significant difference from the untreated control (one-way ANOVA with Dunnett's post hoc test, *p* < 0.0001. Raw viability data from individual experiments are provided in Table S5.

**Figure S2**. Counter-current chromatographic (CCC) separation of monotropein-10-trans-coumarate (peak 2, monitored at 310 nm) and monotropein-10-trans-cinnamate (peak 3, monitored at 280 nm) from the main copigment matrix impurity (peak 1) from extracts of *Gaultheria phillyreifolia* and *G. poeppigii* berries. The separation was performed using a tert-butyl methyl ether/1-butanol/acetonitrile/H₂O (6:1:2:10, v/v/v/v) solvent system acidified with 0.1% trifluoroacetic acid, in head-to-tail mode at 860 rpm. Stationary phase retention was 70.3%. HPLC-DAD analysis of pooled fractions indicated purities of 81.3% and 72.1% for monotropein-10-*trans*-coumarate and monotropein-10-*trans*-cinnamate, respectively.

**Figure S3.** Uptake of monotropein, monotropein-10-coumarate and monotropein-10-cinnamate in the Caco-2:HT29-MTX-E12 co-culture model at different temperatures. Cells were incubated at 4 °C or 37 °C for 1 h in the presence of 100 µmol/L of each test compound. At the end of incubation, cells were washed, quenched and extracted as described in section 2.7. Data are means ± SD of four independent biological replicates (n = 4). Statistical comparisons were performed by two-way ANOVA with Šídák's post hoc test. Monotropein uptake was significantly higher at 4 °C than at 37 °C (p < 0.0001), an observation inconsistent with simple active transport inhibition and likely reflecting reduced efflux or intracellular metabolic activity at lower temperature (see Discussion). No significant temperature effect was observed for monotropein-10-coumarate or monotropein-10-cinnamate (both p > 0.9999).
